# Thrombotic Thrombocytopenic Purpura-Haemolytic Uremic Syndrome and pregnancy

**DOI:** 10.11604/pamj.2014.17.255.2940

**Published:** 2014-04-08

**Authors:** Julius Chacha Mwita, Sandro Vento, Tadele Benti

**Affiliations:** 1 Department of Internal Medicine,University of Botswana and Princess Marina Hospital; 2 Department of Obstetrics &Gynaecology, University of Botswana and Princess Marina Hospital, Botswana

**Keywords:** Thrombotic Thrombocytopenic Purpura-Haemolytic Uremic Syndrome, pregnancy

## Abstract

Thrombotic Thrombocytopenic Purpura-Haemolytic Uremic Syndrome (TTP-HUS) is a rare pregnancy and postpartum complication that may simulate the more common obstetric complications, preeclampsia and the syndrome of haemolysis, elevated liver functions tests, low platelets (HELLP). We describe a 26 years old patient who presented with peri-partum TTP-HUSand was initially treated as a case of HELLP syndrome without any improvement. A brief review of the current TTP-HUS treatment options in pregnancy is also presented.

## Introduction

Thrombotic Thrombocytopenic Purpura-Haemolytic Uremic Syndrome (TTP-HUS) is an infrequent occlusive microangiopathic disorder that is caused by intravascular platelet aggregation. It is strongly associated with a severe deficiency of ADAMTS-13, a metalloprotease that cleaves ultra-large von Willebrand factor (VWF) multimers. The resultant large von Willebrand factor multimers increase platelet adhesiveness and impair fibrinolytic activity with subsequent thrombotic occlusion of the microvasculature [1]. TTP-HUSmay occur for the first time, or be precipitated inlate pregnancy or during the postpartum period for uncertain reasons, as although pregnancy is associated with a physiologic decrease in ADAMTS-13 levels, these remain well above the levels of 5% to 10% that are associated most strongly with TTP-HUS[1]. The clinical presentation of TTP-HUS,consisting of aclassic pentad of microangiopathic haemolytic anaemia, thrombocytopenia, fluctuating central nervous system abnormalities, fever and renal impairment occurs in only about 6 - 40% of patients[2–4]. The triad of anaemia, thrombocytopenia and bizarre neurologic abnormalities can be observed in up to 75% of patients [3, 4]. Because of the inconsistency of non-haematologic findings (fever, neurologic, or renal), the diagnosis of TTP-HUS is often made by a dyad of thrombocytopenia and micro angio-pathichemolyticanemia [3]. All the above clinical presentations overlap with that ofthe more common HELLP syndrome. As the management of the two syndromes differ, an accurate diagnosis is essential.

## Patient and observation

Miss M. is a 26-year-old HIV-negative African (gravida 2, para 0) lady who was booked for antenatal care at 13 weeks gestation. She had a previous abortion because of cervical incompetence. A Shirodkarcerclage was done at 14 weeks gestation. Her antenatal care remained uneventful until 30 weeks plus 2 days gestation when she presented to our hospital with a history of lower abdominal pain, vaginal bleeding and vomiting over the past two hours. Foetal movements had ceased shortly before the above symptoms started. A week before admission she had been diagnosed with right-sided Bell's palsy and started on low dose prednisolone.

On admission, she had a regular pulse rate of 100 beats/minute, a respiratory rate of 28/minute and a blood pressure of 164/105mmHg. Apart from the right sided facial nerve palsy, there were no neurologic deficits. Her skin was normal, devoid of any petechial haemorrhage or ecchymosis. She neither had oedema of the feet nor jaundice. She had a non-tender gravid uterus of about 30 weeks, her cervix was 2cm dilated and 40% effaced and foetal heart beats were absent. Examination findings of the cardiovascular and respiratory systems were unremarkable. Urine dipstick had 2+proteinuria and she was hyperglycemic.

She was diagnosed to have placenta abruption with intrauterine foetal death, preeclampsia, Bell's palsy and gestational diabetes, and was put on methyldopa, nifedipine, hydralazine and magnesium sulphate. A sliding scale of regular insulin was also started, and the Shirodkar suture removed. Four hours after admission she had a vaginal delivery of a fresh still birth weighing 1200gm without any congenital abnormalities. The patient had a postpartum haemorrhage of 550mls, which was treated by uterine massage and oxytocin (10units bolus dose followed by an infusion of 40 units in 500 ml of normal saline). No significant retroplacental clots were noted. Investigations done on admission revealed normocytic anaemia (9. 5gm/dL), leucocytosis (17. 3 x 10^3^/µL) and a platelet count of 201. 6 x 10^3^/µ L of blood. Serum creatinine, urea and liver function tests were within normal limits.

One day after admission she started complaining of fever, palpitations, blurred vision, dizziness, headache and shortness of breath. Physical examination revealed severe conjunctiva pallor, mild pedal oedema, tachypnoea and a clear chest on auscultation. Cardiovascular examination and echocardiographic findings were unremarkable. Her haemoglobin and platelet count had dropped to 6. 5gm/dL and 86 x 10^3^/µL of blood respectively. Two units of packed red cells were transfused and she was started on antibiotics for suspected septicaemia.

On the second day of admission she became anuric with increased facial and bilateral leg swelling. Her laboratory data showed elevated serum creatinine and urea (431µ mol/L and 12. 9mmol/L respectively) and lactate dehydrogenase (1850 IU/L). Her liver enzymes were raised (serum aspartate aminotransferase; 242IU/L and serum alanine aminotransferase; 109IU/L). Based on haemolysis, elevated liver enzymes and thrombocytopenia, HELLP syndrome was diagnosed. A consumptive coagulopathy was also considered because of the clinical presentation of fever and haemolysis that followeda stillbirth. The patient was conservatively treated with whole blood and platelet transfusions. Because of falling urinary output magnesium sulphate was withheld and oral nifedipine continued.

Two days later the patient was still anuric and her creatinine and urea continued to rise. She was confused, pale with petechial haemorrhage and ecchymosis all over her body. Her platelet count had dropped to 35 ×10^3^/56;L. There was no change in her haemoglobin in spite of blood transfusion. She had a normal coagulation profile (Prothrombin time; 9. 8 seconds, Partial thromboplastin time; 25. 4 seconds and international normalized ratio; 0. 96). Serum D-dimer level was 5. 7µg/mL (normal values < 0. 5µg/mL) and, fragmented red blood cells (schistocytes) were found on a peripheral blood smear. In addition, blood cultures were negative.

Finally, we suspected TTP-HUS because of worsening haemolysis (falling haemoglobin level, presence of schistocytes, elevated LDH) and thrombocytopenia in spite of supportive therapy. Consumptive coagulopathy was excluded because of normal coagulation indices. She was, therefore, transferred to the intensive care unit (ICU) and started on dexamethasone, supportive measures and haemodialysis. Platelet transfusions were stopped and she received several units of fresh frozen plasma over subsequent days.

Three weeks after hospital admission the patient's clinical, haematological and biochemical parameters had slowly normalized, and she was discharged home in good clinical condition. Subsequent outpatient visits showed no clinical or biochemical deterioration.

## Discussion

This patient presented with placenta abruption complicated by haemolytic anaemia, thrombocytopenia, elevated transaminases and renal failure. Both the HELLP syndrome and TTP-HUS can present with the clinical picture above. As it was in our case, the more common HELLP syndrome is often diagnosed than the rare pregnancy-related TTP-HUS. Nevertheless, it is important to always consider both diseases as differential diagnoses for a woman presenting with the above features in antenatal or postpartum period. This is not only because of their different treatment options but also for the reason that TTP-HUS is associated with high maternal and foetal mortality rates. The main challenge is how to reliably differentiate the two diseases.

Postpartum HELLP syndrome was the initial diagnosis considered in our patient. HELLP syndrome is a form of severe preeclampsia that may ensue in the postpartum period, usually within 48 hours, in about 30% of cases. Indeed there may be no evidence of preeclampsia before or during labour in 20% of cases[5]. It is characterized by microangiopathichaemolysis, significant elevation of liver enzymes (aspartate aminotransferase and alanine aminotransferase) and a platelet count below 100,000/mm^3^ [6]. The typical clinical symptoms of mid-epigastric or right upper quadrant abdominal pain, nauseaand vomiting occurin 30% to 90% of the cases[7]. Our patient presented with haemolysis, thrombocytopenia and elevated transaminases. She denied any history of epigastric pain. On admission, shehadfeatures of preeclampsia (mild hypertension and proteinuria) which were absent during her antenatalvisits. In addition, she presented with a one week history ofright sided Bell's palsy. Although the aetiology is not clear, there is a strong association between Bell'spalsy and pre-eclampsia[8]; nerve compression due to perineural oedema or increased maternal extracellular volume is the hypothesized explanation. Impending preeclampsia is often considered when Bell'spalsy presents during the third trimester or puerperium [8]. Based on all the above considerations, the present patient was treated for preeclampsia/HELLP syndrome. However, despite of supportive treatment (steroids, platelet and red cell transfusions), her clinical condition deteriorated over the course of her hospital stay. Few days after admission, we finally suspected TTP-HUSon the basis of haemolysis, fever, neurologic symptoms, renal insufficiency and a worsening thrombocytopenia despite delivery, steroid use and transfusion of platelets as well as red cells. While delivery generally leads to a rapid resolution of HELLP syndrome, TTP-HUSworsensif not treated. In view of that, if HELLP syndrome does not improve by 2-3 days postpartum one needs to revise the diagnosis and consider the possibility of Thrombotic Thrombocytopenic Purpura-Haemolytic Uremic Syndrome [4, 9]. Nevertheless, as preeclampsia/HELLP syndromes worsen with more systemic involvement, the distinction from TTP-HUS becomes more challenging and virtually impossible[6]. It should also be noted thatTTP-HUS and preeclampsia/HELLP syndrome may coexist in about 17% of cases [10]. In absence of a straightforward test that unequivocallydistinguishesTTP-HUS from the HELLP syndrome,TTP-HUSremains a clinical diagnosis.

Plasma exchange, immunosuppressive drugs, corticosteroids, nonsteroidal anti-inflammatory drugs, and transfusion of fresh-frozen plasma or packed red blood cells are all used in the treatment of TTP-HUS. Althoughplasma exchange is superior to plasma infusion in the treatment of thrombotic thrombocytopenic purpura, plasma infusion is an appropriate treatment for TTP-HUS patients when plasma exchange is not available or is delayed [10, 11]. TTP-HUS-related maternal mortality can drop from 90% in untreated patients, to less than 10% in women promptly treated with plasma therapy [2, 3, 12]. The effectiveness of plasma therapy has been ascribed to the replacement of ADAMTS 13 activity[11]. Although the decision to begin plasma exchange is not always easy to make, itis appropriate for severe preeclampsia/HELLP syndrome[6], and can be used when spontaneous recovery following delivery is doubtful and clinical worsening or death are likely [6].

While platelet transfusions may be required for HELLP syndrome, administration ofplatelets to a patient with TTP-HUSmay, in contradiction, deteriorate the clinical picture by increasing thrombosis [13]. Therefore platelet transfusion should be avoided and reserved to cases with life-threatening bleeding, such as intracranial haemorrhage[14]. Our patient initially worsened despite platelet transfusions, and dramaticallyimproved after transfusion of fresh frozen plasma. Whereas delivery is the cure for patients withHELLP syndrome, pregnancy can continue inpatients with TTP-HUS. However, the risk of foetaldeath is relatively high due tochronic placental insufficiency that results from maternal hypoxia and/or foetal thrombotic vasculopathy[15]. Serial foetal and maternal ultrasounds are often required for foetal growth monitoring as well as to assess placental blood flow [9]. Periodic plasma infusions may also be neededto ensure adequate levels of ADAMTS13 during pregnancy [9].

## Conclusion

Late in pregnancy, it is difficult to differentiate the common preeclampsia/HELLP syndromes fromTTP-HUS. Both diagnoses should be considered later in the pregnancy and during puerperium. A high index of suspicion is essential for the timely diagnosis and treatment of TTP-HUS. Delivery of the baby is the definitive treatment of severe preeclampsia/HELLP syndrome. If a patient recovers after delivery, TTP-HUS is unlikely. Generally, plasma exchange or infusion of fresh frozen plasma should be considered when a woman presents with TTP-HUS symptoms during puerperium. It should be assertively considered if symptoms and signs persist after delivery. Moreover, if severe thrombocytopenia, haemolytic anaemia, renal failure and mental status changes are present, TTP-HUS should be diagnosed and treated with plasma exchange or fresh frozen plasma, regardless of the time of the pregnancy.
